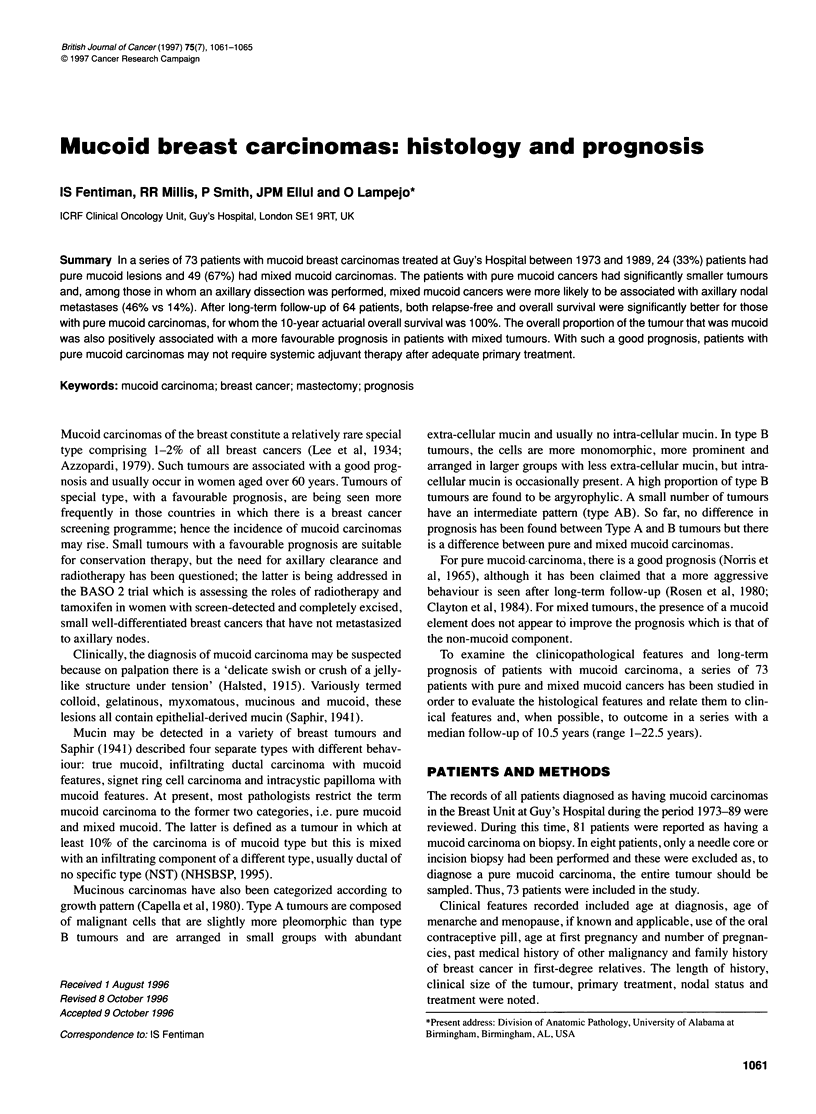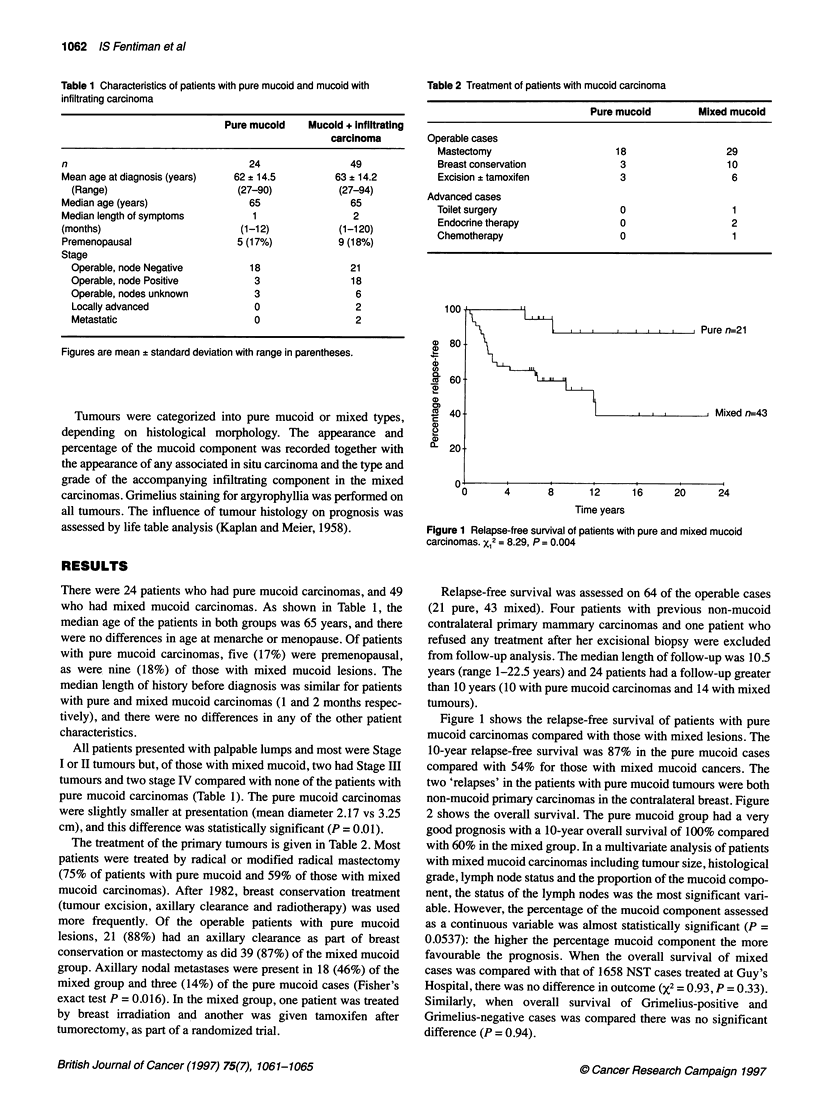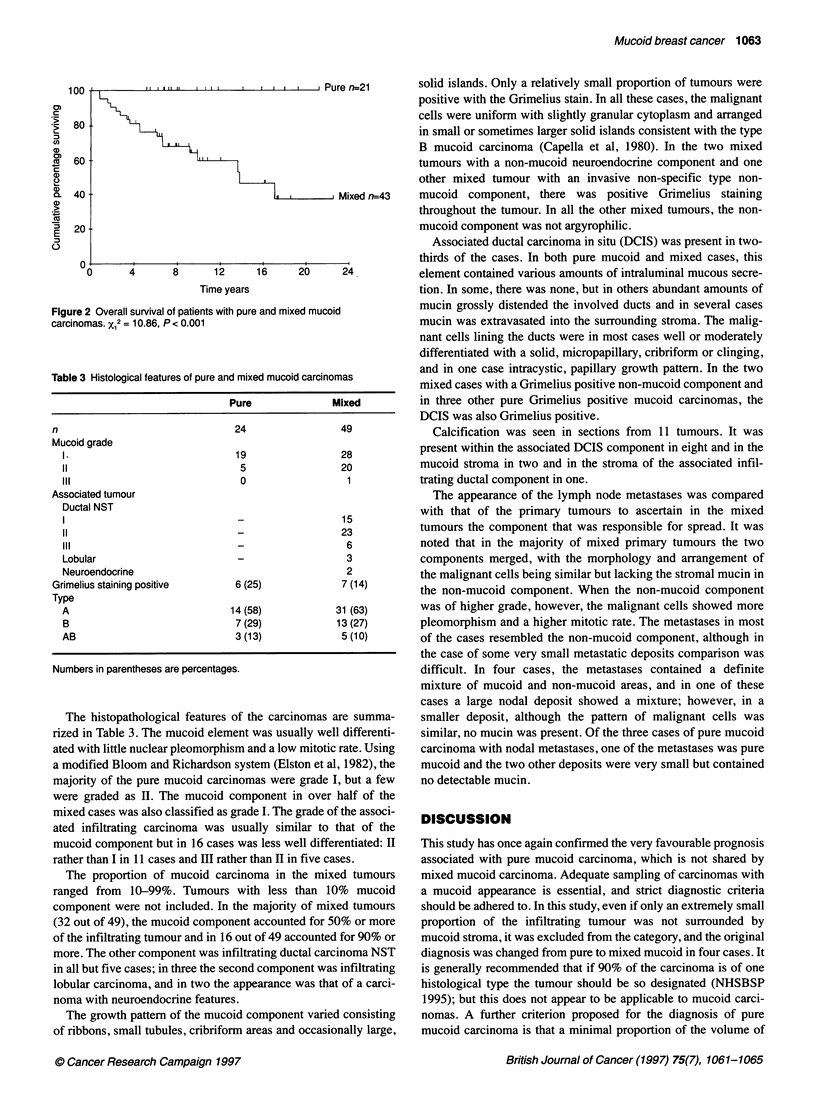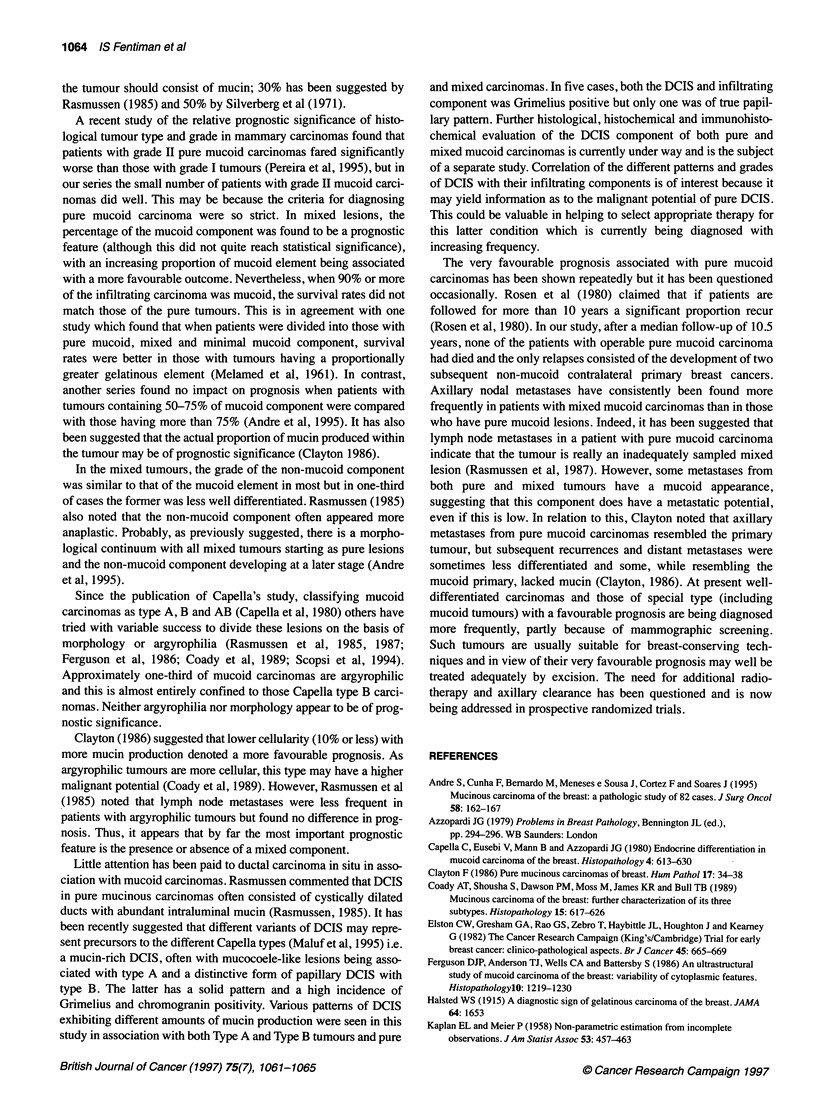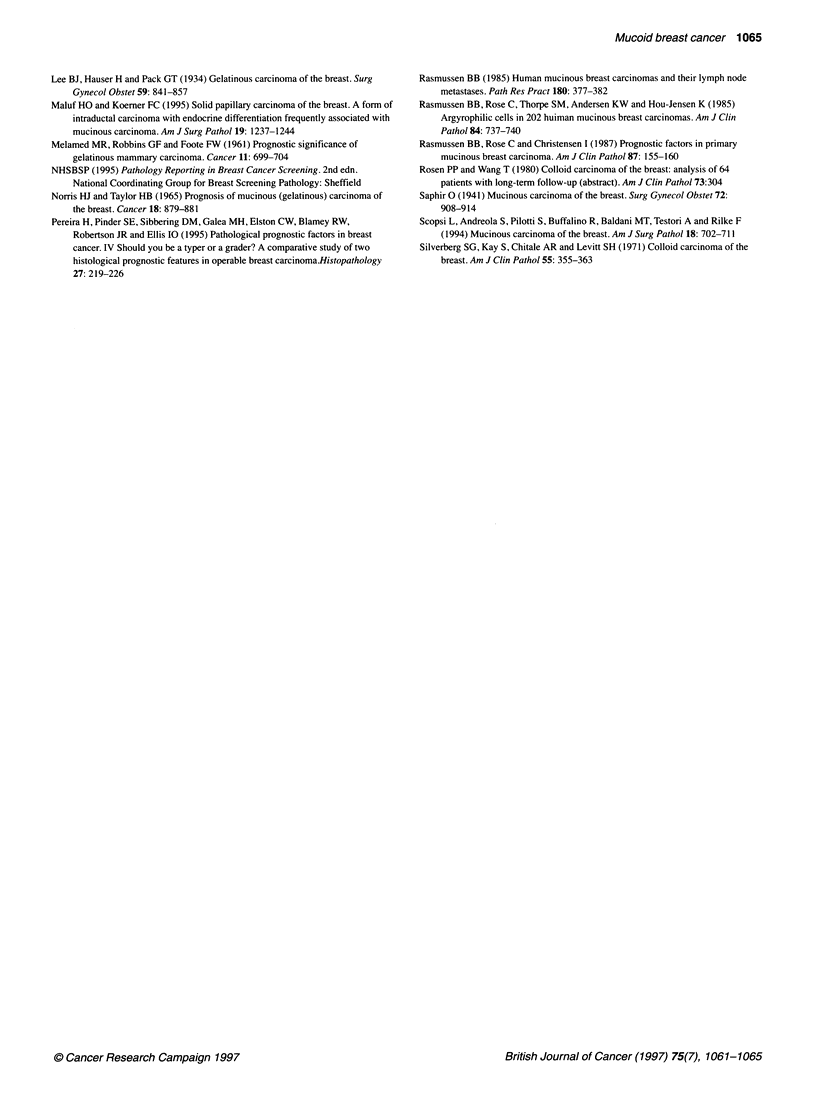# Mucoid breast carcinomas: histology and prognosis.

**DOI:** 10.1038/bjc.1997.180

**Published:** 1997

**Authors:** I. S. Fentiman, R. R. Millis, P. Smith, J. P. Ellul, O. Lampejo

**Affiliations:** ICRF Clinical Oncology Unit, Guy's Hospital, London, UK.

## Abstract

In a series of 73 patients with mucoid breast carcinomas treated at Guy's Hospital between 1973 and 1989, 24 (33%) patients had pure mucoid lesions and 49 (67%) had mixed mucoid carcinomas. The patients with pure mucoid cancers had significantly smaller tumours and, among those in whom an axillary dissection was performed, mixed mucoid cancers were more likely to be associated with axillary nodal metastases (46% vs 14%). After long-term follow-up of 64 patients, both relapse-free and overall survival were significantly better for those with pure mucoid carcinomas, for whom the 10-year actuarial overall survival was 100%. The overall proportion of the tumour that was mucoid was also positively associated with a more favourable prognosis in patients with mixed tumours. With such a good prognosis, patients with pure mucoid carcinomas may not require systemic adjuvant therapy after adequate primary treatment.


British Journal of Cancer (1997) 75(7), 1061–1065
© 1997 Cancer Research Campaign

### Mucoid breast carcinomas: histology and prognosis

**IS Fentiman, RR Millis, P Smith, JPM Ellul and O Lampejo***

ICRF Clinical Oncology Unit, Guy's Hospital, London SE1 9RT, UK

**Summary** In a series of 73 patients with mucoid breast carcinomas treated at Guy's Hospital between 1973 and 1989, 24 (33%) patients had pure mucoid lesions and 49 (67%) had mixed mucoid carcinomas. The patients with pure mucoid cancers had significantly smaller tumours and, among those in whom an axillary dissection was performed, mixed mucoid cancers were more likely to be associated with axillary nodal metastases (46% vs 14%). After long-term follow-up of 64 patients, both relapse-free and overall survival were significantly better for those with pure mucoid carcinomas, for whom the 10-year actuarial overall survival was 100%. The overall proportion of the tumour that was mucoid was also positively associated with a more favourable prognosis in patients with mixed tumours. With such a good prognosis, patients with pure mucoid carcinomas may not require systemic adjuvant therapy after adequate primary treatment.

**Keywords:** mucoid carcinoma; breast cancer; mastectomy; prognosis

Mucoid carcinomas of the breast constitute a relatively rare special type comprising 1–2% of all breast cancers (Lee et al, 1934; Azzopardi, 1979). Such tumours are associated with a good prognosis and usually occur in women aged over 60 years. Tumours of special type, with a favourable prognosis, are being seen more frequently in those countries in which there is a breast cancer screening programme; hence the incidence of mucoid carcinomas may rise. Small tumours with a favourable prognosis are suitable for conservation therapy, but the need for axillary clearance and radiotherapy has been questioned; the latter is being addressed in the BASO 2 trial which is assessing the roles of radiotherapy and tamoxifen in women with screen-detected and completely excised, small well-differentiated breast cancers that have not metastasized to axillary nodes.

Clinically, the diagnosis of mucoid carcinoma may be suspected because on palpation there is a 'delicate swish or crush of a jelly-like structure under tension' (Halsted, 1915). Variously termed colloid, gelatinous, myxomatous, mucinous and mucoid, these lesions all contain epithelial-derived mucin (Saphir, 1941).

Mucin may be detected in a variety of breast tumours and Saphir (1941) described four separate types with different behaviour: true mucoid, infiltrating ductal carcinoma with mucoid features, signet ring cell carcinoma and intracystic papilloma with mucoid features. At present, most pathologists restrict the term mucoid carcinoma to the former two categories, i.e. pure mucoid and mixed mucoid. The latter is defined as a tumour in which at least 10% of the carcinoma is of mucoid type but this is mixed with an infiltrating component of a different type, usually ductal of no specific type (NST) (NHSBSP, 1995).

Mucinous carcinomas have also been categorized according to growth pattern (Capella et al, 1980). Type A tumours are composed of malignant cells that are slightly more pleomorphic than type B tumours and are arranged in small groups with abundant extra-cellular mucin and usually no intra-cellular mucin. In type B tumours, the cells are more monomorphic, more prominent and arranged in larger groups with less extra-cellular mucin, but intra-cellular mucin is occasionally present. A high proportion of type B tumours are found to be argyrophylic. A small number of tumours have an intermediate pattern (type AB). So far, no difference in prognosis has been found between Type A and B tumours but there is a difference between pure and mixed mucoid carcinomas.

For pure mucoid carcinoma, there is a good prognosis (Norris et al, 1965), although it has been claimed that a more aggressive behaviour is seen after long-term follow-up (Rosen et al, 1980; Clayton et al, 1984). For mixed tumours, the presence of a mucoid element does not appear to improve the prognosis which is that of the non-mucoid component.

To examine the clinicopathological features and long-term prognosis of patients with mucoid carcinoma, a series of 73 patients with pure and mixed mucoid cancers has been studied in order to evaluate the histological features and relate them to clinical features and, when possible, to outcome in a series with a median follow-up of 10.5 years (range 1–22.5 years).

### PATIENTS AND METHODS

The records of all patients diagnosed as having mucoid carcinomas in the Breast Unit at Guy's Hospital during the period 1973–89 were reviewed. During this time, 81 patients were reported as having a mucoid carcinoma on biopsy. In eight patients, only a needle core or incision biopsy had been performed and these were excluded as, to diagnose a pure mucoid carcinoma, the entire tumour should be sampled. Thus, 73 patients were included in the study.

Clinical features recorded included age at diagnosis, age of menarche and menopause, if known and applicable, use of the oral contraceptive pill, age at first pregnancy and number of pregnancies, past medical history of other malignancy and family history of breast cancer in first-degree relatives. The length of history, clinical size of the tumour, primary treatment, nodal status and treatment were noted.

*Received 1 August 1996*
*Revised 8 October 1996*
*Accepted 9 October 1996*

*Correspondence to:* IS Fentiman

*Present address: Division of Anatomic Pathology, University of Alabama at Birmingham, Birmingham, AL, USA

**Table 1** Characteristics of patients with pure mucoid and mucoid with infiltrating carcinoma

| | Pure mucoid | Mucoid + infiltrating carcinoma |
|---|---|---|
| *n* | 24 | 49 |
| Mean age at diagnosis (years) | 62 ± 14.5 | 63 ± 14.2 |
| (Range) | (27–90) | (27–94) |
| Median age (years) | 65 | 65 |
| Median length of symptoms (months) | 1 (1–12) | 2 (1–120) |
| Premenopausal | 5 (17%) | 9 (18%) |
| Stage | | |
| Operable, node Negative | 18 | 21 |
| Operable, node Positive | 3 | 18 |
| Operable, nodes unknown | 3 | 6 |
| Locally advanced | 0 | 2 |
| Metastatic | 0 | 2 |

Figures are mean ± standard deviation with range in parentheses.

Tumours were categorized into pure mucoid or mixed types, depending on histological morphology. The appearance and percentage of the mucoid component was recorded together with the appearance of any associated in situ carcinoma and the type and grade of the accompanying infiltrating component in the mixed carcinomas. Grimelius staining for argyrophyllia was performed on all tumours. The influence of tumour histology on prognosis was assessed by life table analysis (Kaplan and Meier, 1958).

### RESULTS

There were 24 patients who had pure mucoid carcinomas, and 49 who had mixed mucoid carcinomas. As shown in Table 1, the median age of the patients in both groups was 65 years, and there were no differences in age at menarche or menopause. Of patients with pure mucoid carcinomas, five (17%) were premenopausal, as were nine (18%) of those with mixed mucoid lesions. The median length of history before diagnosis was similar for patients with pure and mixed mucoid carcinomas (1 and 2 months respectively), and there were no differences in any of the other patient characteristics.

All patients presented with palpable lumps and most were Stage I or II tumours but, of those with mixed mucoid, two had Stage III tumours and two stage IV compared with none of the patients with pure mucoid carcinomas (Table 1). The pure mucoid carcinomas were slightly smaller at presentation (mean diameter 2.17 vs 3.25 cm), and this difference was statistically significant ($P = 0.01$).

The treatment of the primary tumours is given in Table 2. Most patients were treated by radical or modified radical mastectomy (75% of patients with pure mucoid and 59% of those with mixed mucoid carcinomas). After 1982, breast conservation treatment (tumour excision, axillary clearance and radiotherapy) was used more frequently. Of the operable patients with pure mucoid lesions, 21 (88%) had an axillary clearance as part of breast conservation or mastectomy as did 39 (87%) of the mixed mucoid group. Axillary nodal metastases were present in 18 (46%) of the mixed group and three (14%) of the pure mucoid cases (Fisher's exact test $P = 0.016$). In the mixed group, one patient was treated by breast irradiation and another was given tamoxifen after tumorectomy, as part of a randomized trial.

**Table 2** Treatment of patients with mucoid carcinoma

| | Pure mucoid | Mixed mucoid |
|---|---|---|
| Operable cases | | |
| Mastectomy | 18 | 29 |
| Breast conservation | 3 | 10 |
| Excision ± tamoxifen | 3 | 6 |
| Advanced cases | | |
| Toilet surgery | 0 | 1 |
| Endocrine therapy | 0 | 2 |
| Chemotherapy | 0 | 1 |

**Figure 1** Relapse-free survival of patients with pure and mixed mucoid carcinomas. $\chi_1^2 = 8.29$, $P = 0.004$

Relapse-free survival was assessed on 64 of the operable cases (21 pure, 43 mixed). Four patients with previous non-mucoid contralateral primary mammary carcinomas and one patient who refused any treatment after her excisional biopsy were excluded from follow-up analysis. The median length of follow-up was 10.5 years (range 1–22.5 years) and 24 patients had a follow-up greater than 10 years (10 with pure mucoid carcinomas and 14 with mixed tumours).

Figure 1 shows the relapse-free survival of patients with pure mucoid carcinomas compared with those with mixed lesions. The 10-year relapse-free survival was 87% in the pure mucoid cases compared with 54% for those with mixed mucoid cancers. The two 'relapses' in the patients with pure mucoid tumours were both non-mucoid primary carcinomas in the contralateral breast. Figure 2 shows the overall survival. The pure mucoid group had a very good prognosis with a 10-year overall survival of 100% compared with 60% in the mixed group. In a multivariate analysis of patients with mixed mucoid carcinomas including tumour size, histological grade, lymph node status and the proportion of the mucoid component, the status of the lymph nodes was the most significant variable. However, the percentage of the mucoid component assessed as a continuous variable was almost statistically significant ($P = 0.0537$): the higher the percentage mucoid component the more favourable the prognosis. When the overall survival of mixed cases was compared with that of 1658 NST cases treated at Guy's Hospital, there was no difference in outcome ($\chi^2 = 0.93$, $P = 0.33$). Similarly, when overall survival of Grimelius-positive and Grimelius-negative cases was compared there was no significant difference ($P = 0.94$).

© Cancer Research Campaign 1997

**Figure 2** Overall survival of patients with pure and mixed mucoid carcinomas. $\chi_1^2 = 10.86$, $P < 0.001$

**Table 3** Histological features of pure and mixed mucoid carcinomas

| | Pure | Mixed |
|---|---|---|
| *n* | 24 | 49 |
| Mucoid grade | | |
| I | 19 | 28 |
| II | 5 | 20 |
| III | 0 | 1 |
| Associated tumour | | |
| Ductal NST | | |
| I | – | 15 |
| II | – | 23 |
| III | – | 6 |
| Lobular | – | 3 |
| Neuroendocrine | | 2 |
| Grimelius staining positive | 6 (25) | 7 (14) |
| Type | | |
| A | 14 (58) | 31 (63) |
| B | 7 (29) | 13 (27) |
| AB | 3 (13) | 5 (10) |

Numbers in parentheses are percentages.

The histopathological features of the carcinomas are summarized in Table 3. The mucoid element was usually well differentiated with little nuclear pleomorphism and a low mitotic rate. Using a modified Bloom and Richardson system (Elston et al, 1982), the majority of the pure mucoid carcinomas were grade I, but a few were graded as II. The mucoid component in over half of the mixed cases was also classified as grade I. The grade of the associated infiltrating carcinoma was usually similar to that of the mucoid component but in 16 cases was less well differentiated: II rather than I in 11 cases and III rather than II in five cases.

The proportion of mucoid carcinoma in the mixed tumours ranged from 10–99%. Tumours with less than 10% mucoid component were not included. In the majority of mixed tumours (32 out of 49), the mucoid component accounted for 50% or more of the infiltrating tumour and in 16 out of 49 accounted for 90% or more. The other component was infiltrating ductal carcinoma NST in all but five cases; in three the second component was infiltrating lobular carcinoma, and in two the appearance was that of a carcinoma with neuroendocrine features.

The growth pattern of the mucoid component varied consisting of ribbons, small tubules, cribriform areas and occasionally large, solid islands. Only a relatively small proportion of tumours were positive with the Grimelius stain. In all these cases, the malignant cells were uniform with slightly granular cytoplasm and arranged in small or sometimes larger solid islands consistent with the type B mucoid carcinoma (Capella et al, 1980). In the two mixed tumours with a non-mucoid neuroendocrine component and one other mixed tumour with an invasive non-specific type non-mucoid component, there was positive Grimelius staining throughout the tumour. In all the other mixed tumours, the non-mucoid component was not argyrophilic.

Associated ductal carcinoma in situ (DCIS) was present in two-thirds of the cases. In both pure mucoid and mixed cases, this element contained various amounts of intraluminal mucous secretion. In some, there was none, but in others abundant amounts of mucin grossly distended the involved ducts and in several cases mucin was extravasated into the surrounding stroma. The malignant cells lining the ducts were in most cases well or moderately differentiated with a solid, micropapillary, cribriform or clinging, and in one case intracystic, papillary growth pattern. In the two mixed cases with a Grimelius positive non-mucoid component and in three other pure Grimelius positive mucoid carcinomas, the DCIS was also Grimelius positive.

Calcification was seen in sections from 11 tumours. It was present within the associated DCIS component in eight and in the mucoid stroma in two and in the stroma of the associated infiltrating ductal component in one.

The appearance of the lymph node metastases was compared with that of the primary tumours to ascertain in the mixed tumours the component that was responsible for spread. It was noted that in the majority of mixed primary tumours the two components merged, with the morphology and arrangement of the malignant cells being similar but lacking the stromal mucin in the non-mucoid component. When the non-mucoid component was of higher grade, however, the malignant cells showed more pleomorphism and a higher mitotic rate. The metastases in most of the cases resembled the non-mucoid component, although in the case of some very small metastatic deposits comparison was difficult. In four cases, the metastases contained a definite mixture of mucoid and non-mucoid areas, and in one of these cases a large nodal deposit showed a mixture; however, in a smaller deposit, although the pattern of malignant cells was similar, no mucin was present. Of the three cases of pure mucoid carcinoma with nodal metastases, one of the metastases was pure mucoid and the two other deposits were very small but contained no detectable mucin.

### DISCUSSION

This study has once again confirmed the very favourable prognosis associated with pure mucoid carcinoma, which is not shared by mixed mucoid carcinoma. Adequate sampling of carcinomas with a mucoid appearance is essential, and strict diagnostic criteria should be adhered to. In this study, even if only an extremely small proportion of the infiltrating tumour was not surrounded by mucoid stroma, it was excluded from the category, and the original diagnosis was changed from pure to mixed mucoid in four cases. It is generally recommended that if 90% of the carcinoma is of one histological type the tumour should be so designated (NHSBSP 1995); but this does not appear to be applicable to mucoid carcinomas. A further criterion proposed for the diagnosis of pure mucoid carcinoma is that a minimal proportion of the volume of

© Cancer Research Campaign 1997

the tumour should consist of mucin; 30% has been suggested by Rasmussen (1985) and 50% by Silverberg et al (1971).

A recent study of the relative prognostic significance of histological tumour type and grade in mammary carcinomas found that patients with grade II pure mucoid carcinomas fared significantly worse than those with grade I tumours (Pereira et al, 1995), but in our series the small number of patients with grade II mucoid carcinomas did well. This may be because the criteria for diagnosing pure mucoid carcinoma were so strict. In mixed lesions, the percentage of the mucoid component was found to be a prognostic feature (although this did not quite reach statistical significance), with an increasing proportion of mucoid element being associated with a more favourable outcome. Nevertheless, when 90% or more of the infiltrating carcinoma was mucoid, the survival rates did not match those of the pure tumours. This is in agreement with one study which found that when patients were divided into those with pure mucoid, mixed and minimal mucoid component, survival rates were better in those with tumours having a proportionally greater gelatinous element (Melamed et al, 1961). In contrast, another series found no impact on prognosis when patients with tumours containing 50–75% of mucoid component were compared with those having more than 75% (Andre et al, 1995). It has also been suggested that the actual proportion of mucin produced within the tumour may be of prognostic significance (Clayton 1986).

In the mixed tumours, the grade of the non-mucoid component was similar to that of the mucoid element in most but in one-third of cases the former was less well differentiated. Rasmussen (1985) also noted that the non-mucoid component often appeared more anaplastic. Probably, as previously suggested, there is a morphological continuum with all mixed tumours starting as pure lesions and the non-mucoid component developing at a later stage (Andre et al, 1995).

Since the publication of Capella's study, classifying mucoid carcinomas as type A, B and AB (Capella et al, 1980) others have tried with variable success to divide these lesions on the basis of morphology or argyrophilia (Rasmussen et al, 1985, 1987; Ferguson et al, 1986; Coady et al, 1989; Scopsi et al, 1994). Approximately one-third of mucoid carcinomas are argyrophilic and this is almost entirely confined to those Capella type B carcinomas. Neither argyrophilia nor morphology appear to be of prognostic significance.

Clayton (1986) suggested that lower cellularity (10% or less) with more mucin production denoted a more favourable prognosis. As argyrophilic tumours are more cellular, this type may have a higher malignant potential (Coady et al, 1989). However, Rasmussen et al (1985) noted that lymph node metastases were less frequent in patients with argyrophilic tumours but found no difference in prognosis. Thus, it appears that by far the most important prognostic feature is the presence or absence of a mixed component.

Little attention has been paid to ductal carcinoma in situ in association with mucoid carcinomas. Rasmussen commented that DCIS in pure mucinous carcinomas often consisted of cystically dilated ducts with abundant intraluminal mucin (Rasmussen, 1985). It has been recently suggested that different variants of DCIS may represent precursors to the different Capella types (Maluf et al, 1995) i.e. a mucin-rich DCIS, often with mucocoele-like lesions being associated with type A and a distinctive form of papillary DCIS with type B. The latter has a solid pattern and a high incidence of Grimelius and chromogranin positivity. Various patterns of DCIS exhibiting different amounts of mucin production were seen in this study in association with both Type A and Type B tumours and pure and mixed carcinomas. In five cases, both the DCIS and infiltrating component was Grimelius positive but only one was of true papillary pattern. Further histological, histochemical and immunohistochemical evaluation of the DCIS component of both pure and mixed mucoid carcinomas is currently under way and is the subject of a separate study. Correlation of the different patterns and grades of DCIS with their infiltrating components is of interest because it may yield information as to the malignant potential of pure DCIS. This could be valuable in helping to select appropriate therapy for this latter condition which is currently being diagnosed with increasing frequency.

The very favourable prognosis associated with pure mucoid carcinomas has been shown repeatedly but it has been questioned occasionally. Rosen et al (1980) claimed that if patients are followed for more than 10 years a significant proportion recur (Rosen et al, 1980). In our study, after a median follow-up of 10.5 years, none of the patients with operable pure mucoid carcinoma had died and the only relapses consisted of the development of two subsequent non-mucoid contralateral primary breast cancers. Axillary nodal metastases have consistently been found more frequently in patients with mixed mucoid carcinomas than in those who have pure mucoid lesions. Indeed, it has been suggested that lymph node metastases in a patient with pure mucoid carcinoma indicate that the tumour is really an inadequately sampled mixed lesion (Rasmussen et al, 1987). However, some metastases from both pure and mixed tumours have a mucoid appearance, suggesting that this component does have a metastatic potential, even if this is low. In relation to this, Clayton noted that axillary metastases from pure mucoid carcinomas resembled the primary tumour, but subsequent recurrences and distant metastases were sometimes less differentiated and some, while resembling the mucoid primary, lacked mucin (Clayton, 1986). At present well-differentiated carcinomas and those of special type (including mucoid tumours) with a favourable prognosis are being diagnosed more frequently, partly because of mammographic screening. Such tumours are usually suitable for breast-conserving techniques and in view of their very favourable prognosis may well be treated adequately by excision. The need for additional radiotherapy and axillary clearance has been questioned and is now being addressed in prospective randomized trials.

### REFERENCES

Andre S, Cunha F, Bernardo M, Meneses e Sousa J, Cortez F and Soares J (1995) Mucinous carcinoma of the breast: a pathologic study of 82 cases. *J Surg Oncol* **58**: 162–167

Azzopardi JG (1979) *Problems in Breast Pathology*, Bennington JL (ed.), pp. 294–296. WB Saunders: London

Capella C, Eusebi V, Mann B and Azzopardi JG (1980) Endocrine differentiation in mucoid carcinoma of the breast. *Histopathology* **4**: 613–630

Clayton F (1986) Pure mucinous carcinomas of breast. *Hum Pathol* **17**: 34–38

Coady AT, Shousha S, Dawson PM, Moss M, James KR and Bull TB (1989) Mucinous carcinoma of the breast: further characterization of its three subtypes. *Histopathology* **15**: 617–626

Elston CW, Gresham GA, Rao GS, Zebro T, Haybittle JL, Houghton J and Kearney G (1982) The Cancer Research Campaign (King's/Cambridge) Trial for early breast cancer: clinico-pathological aspects. *Br J Cancer* **45**: 665–669

Ferguson DJP, Anderson TJ, Wells CA and Battersby S (1986) An ultrastructural study of mucoid carcinoma of the breast: variability of cytoplasmic features. *Histopathology***10**: 1219–1230

Halsted WS (1915) A diagnostic sign of gelatinous carcinoma of the breast. *JAMA* **64**: 1653

Kaplan EL and Meier P (1958) Non-parametric estimation from incomplete observations. *J Am Statist Assoc* **53**: 457–463

*© Cancer Research Campaign 1997*

Lee BJ, Hauser H and Pack GT (1934) Gelatinous carcinoma of the breast. *Surg Gynecol Obstet* **59**: 841–857
Maluf HO and Koerner FC (1995) Solid papillary carcinoma of the breast. A form of intraductal carcinoma with endocrine differentiation frequently associated with mucinous carcinoma. *Am J Surg Pathol* **19**: 1237–1244
Melamed MR, Robbins GF and Foote FW (1961) Prognostic significance of gelatinous mammary carcinoma. *Cancer* **11**: 699–704
NHSBSP (1995) *Pathology Reporting in Breast Cancer Screening*. 2nd edn. National Coordinating Group for Breast Screening Pathology: Sheffield
Norris HJ and Taylor HB (1965) Prognosis of mucinous (gelatinous) carcinoma of the breast. *Cancer* **18**: 879–881
Pereira H, Pinder SE, Sibbering DM, Galea MH, Elston CW, Blamey RW, Robertson JR and Ellis IO (1995) Pathological prognostic factors in breast cancer. IV Should you be a typer or a grader? A comparative study of two histological prognostic features in operable breast carcinoma.*Histopathology* **27**: 219–226
Rasmussen BB (1985) Human mucinous breast carcinomas and their lymph node metastases. *Path Res Pract* **180**: 377–382
Rasmussen BB, Rose C, Thorpe SM, Andersen KW and Hou-Jensen K (1985) Argyrophilic cells in 202 huiman mucinous breast carcinomas. *Am J Clin Pathol* **84**: 737–740
Rasmussen BB, Rose C and Christensen I (1987) Prognostic factors in primary mucinous breast carcinoma. *Am J Clin Pathol* **87**: 155–160
Rosen PP and Wang T (1980) Colloid carcinoma of the breast: analysis of 64 patients with long-term follow-up (abstract). *Am J Clin Pathol* **73**:304
Saphir O (1941) Mucinous carcinoma of the breast. *Surg Gynecol Obstet* **72**: 908–914
Scopsi L, Andreola S, Pilotti S, Buffalino R, Baldani MT, Testori A and Rilke F (1994) Mucinous carcinoma of the breast. *Am J Surg Pathol* **18**: 702–711
Silverberg SG, Kay S, Chitale AR and Levitt SH (1971) Colloid carcinoma of the breast. *Am J Clin Pathol* **55**: 355–363

© Cancer Research Campaign 1997